# Seven Years at High Salinity—Experimental Evolution of the Extremely Halotolerant Black Yeast *Hortaea werneckii*

**DOI:** 10.3390/jof7090723

**Published:** 2021-09-04

**Authors:** Cene Gostinčar, Jason E. Stajich, Anja Kejžar, Sunita Sinha, Corey Nislow, Metka Lenassi, Nina Gunde-Cimerman

**Affiliations:** 1 Department of Biology, Biotechnical Faculty, University of Ljubljana, Jamnikarjeva 101, SI-1000 Ljubljana, Slovenia; nina.gunde-cimerman@bf.uni-lj.si; 2 Department of Plant Pathology and Microbiology, Institute for Integrative Genome Biology, University of California, Riverside, CA 92521, USA; jason.stajich@ucr.edu; 3 Institute of Biochemistry and Molecular Genetics, Faculty of Medicine, University of Ljubljana, Vrazov trg 2, SI-1000 Ljubljana, Slovenia; anja.kejzar@mf.uni-lj.si (A.K.); metka.lenassi@mf.uni-lj.si (M.L.); 4 Faculty of Pharmaceutical Sciences, University of British Columbia, Vancouver, BC V6T 1Z3, Canada; sunita.sinha@ubc.ca (S.S.); corey.nislow@utoronto.ca (C.N.)

**Keywords:** experimental evolution, fungi, high salinity, halotolerance, aneuploidy

## Abstract

The experimental evolution of microorganisms exposed to extreme conditions can provide insight into cellular adaptation to stress. Typically, stress-sensitive species are exposed to stress over many generations and then examined for improvements in their stress tolerance. In contrast, when starting with an already stress-tolerant progenitor there may be less room for further improvement, it may still be able to tweak its cellular machinery to increase extremotolerance, perhaps at the cost of poorer performance under non-extreme conditions. To investigate these possibilities, a strain of extremely halotolerant black yeast *Hortaea werneckii* was grown for over seven years through at least 800 generations in a medium containing 4.3 M NaCl. Although this salinity is well above the optimum (0.8–1.7 M) for the species, the growth rate of the evolved *H. werneckii* did not change in the absence of salt or at high concentrations of NaCl, KCl, sorbitol, or glycerol. Other phenotypic traits did change during the course of the experimental evolution, including fewer multicellular chains in the evolved strains, significantly narrower cells, increased resistance to caspofungin, and altered melanisation. Whole-genome sequencing revealed the occurrence of multiple aneuploidies during the experimental evolution of the otherwise diploid *H. werneckii*. A significant overrepresentation of several gene groups was observed in aneuploid regions. Taken together, these changes suggest that long-term growth at extreme salinity led to alterations in cell wall and morphology, signalling pathways, and the pentose phosphate cycle. Although there is currently limited evidence for the adaptive value of these changes, they offer promising starting points for future studies of fungal halotolerance.

## 1. Introduction

With their short generation times and large populations, microorganisms adapt so rapidly that their evolution can be observed on timescales practically achievable in laboratory settings. Microbial cells do not have the protection enjoyed by the cells of multicellular organisms. Combined with their limited ability to ameliorate or escape hostile environmental conditions, rapid adaptation is one of the most important strategies for microbial fitness, competitiveness, and survival. Adaptations may occur at both the phenotypic and genotypic levels and both can be studied to determine both the principles of evolution and the mechanisms of adaptation.

The first attempt at experimental microbial evolution was made as early as the 19th century [1]. However, the most famous experiment, which has now been running for over three decades, was started by Lenski et al. [2]. In this experiment, 12 *Escherichia coli* populations derived from the same progenitor are transferred each day into a fresh minimal liquid medium. The experiment showed how quickly microorganisms can adapt and diversify even under rather homogenous, spatially unstructured, and modestly stressful conditions [3]. The fitness of these evolved strains increased initially in large steps, then more slowly. Modelling suggested that the increase in fitness follows a power-law model and thus theoretically improves in smaller and smaller increments with no limit. In practice, populations diversified, most lineages evolved towards extinction during the experiment, some evolved unexpected metabolic traits, and some even found complementary niches. Hypermutability evolved in six populations and aerobic growth on citrate occurred in a single strain [3]. Since the beginning of the Lenski experiment, experimental evolution has become a powerful tool for studying adaptation and evolution, especially when combined with new high-throughput methods for studying the evolved strains, such as next-generation sequencing technologies.

In fungi, most experimental evolution studies have been performed with *S. cerevisiae*. Gerstein et al. [4] observed that haploid and tetraploid strains of this species tend to converge to diploidy and this occurs more rapidly in haploid strains at high salinity. Ratcliff et al. [5] studied the evolution of multicellularity. Lang et al. [6] explored the rise and fall of polymorphisms in a population. Levy et al. [7] used barcodes to track the relative abundances of about half a million lineages in a population, and showed that beneficial mutations with small effects reproducibly allow transient overgrowth of some lineages before being outcompeted by lineages carrying rarer mutations with large effects in a more stochastic manner. Kozela and Johnston [8] examined the effects of growth-limiting salt stress on mutational variation and the mutation rate. Johnson et al. [9] performed a large-scale evolutionary experiment in three environments and identified several features of adaptation thought to be common across many species. Other fungal species are rarely used in experimental evolution, although fungi, especially microfungi, are generally good experimental subjects due to their relatively short generation times, compact genomes, and large effective population sizes compared to other eukaryotes. The few fungi that have been used for experimental evolution in the last two decades include species in the genera *Aspergillus*, *Candida*, *Neurospora*, and *Schizophyllum* [10].

One of the topics most tractable for experimental evolutionary studies is stress tolerance. Adaptation to extreme conditions is studied for various environmental parameters, from extreme temperatures, salinity, pH, radiation, and toxic chemicals. In addition to this basic research, applied evolutionary engineering for improved stress tolerance also provides insights into modes of adaptation to stress. For example, *Serratia marcescens* improved its salt tolerance to a similar level when evolved at 8% or 10% NaCl (*w*/*v*) despite differences in stress severity [11]; in *Desulfovibrio vulgaris* the increased salt tolerance was attributed to increases in intracellular glutamate and one of the phospholipid fatty acids [12]; in *S. cerevisiae* grown under high salinity, a cluster of mutations in the proton exporter gene PMA1 and expansions of the sodium transport ATPase ENA gene cluster were reported [13]. Other authors observed modest changes in basal expression and regulation of nearly 150 genes in evolved strains of *S. cerevisiae*, as well as an increase in genome size [14]. In contrast, the evolution of *S. cerevisiae* in a medium containing high concentrations of sorbitol and NaCl at 12 °C to improve freezing tolerance resulted in a strain with increased NaCl tolerance but reduced genome size [15]. Finally, a more halotolerant *S. cerevisiae* strain obtained by chemical mutagenesis and culturing in the presence of increasing NaCl concentrations was found to have upregulated expression of genes related to stress response, carbohydrate transport, glycogen and trehalose biosynthesis, and biofilm formation [16].

Experimental evolution under extreme conditions typically involves the study of mesophilic microorganisms to explore the improvements in their ability to cope with stressful conditions. The dynamic range for improvement in such species is large, but their genetic repertoire associated with stress tolerance available for evolutionary tinkering is likely to be limited. We hypothesize that the opposite would be true for an extremotolerant species exposed to extreme conditions that it already copes with reasonably efficiently, i.e., while the genomic resources for adaptation may be large the dynamic range for improvement over baseline is likely limited. Would such a species thrive without major changes, or would it continue to adapt, optimize its mechanisms for coping with stress, and improve its fitness, possibly indefinitely, as suggested by the Lenski experiment [17]?

To address this question experimentally, we used *Hortaea werneckii* (Capnodiales, Dothideomycetidae, Dothideomycetes, Pezizomycotina, Ascomycota), an ascomycetous black yeast that can grow in media nearly saturated with NaCl [18]. Nevertheless, it is classified as extremely halotolerant and not halophilic to acknowledge its ability to thrive in standard media without salt addition. This salinity growth range is outstanding but appears to be little used in the wild, as the species rarely occurs in habitats without at least moderate amounts of salt [19]. The optimal salinity for its growth is between 0.8 M and 1.7 M NaCl [20]. Several studies have addressed the molecular adaptations of *H. werneckii* to hypersaline conditions, from the synthesis of compatible solutes and the use of numerous alkali-metal cation transporters to changes in cell membrane lipid composition, changes in cell wall structure, and the use of the high-osmolarity glycerol pathway (HOG; reviewed in Plemenitaš et al. [20]). The whole genome of *H. werneckii* was sequenced [21,22], and a comparison of different wild isolates revealed that most strains are diploid and highly heterozygous, possibly originating from rare hybridizations between haploid progenitors, but otherwise showing no signs of sexual reproduction [23]. This was later confirmed by whole-genome sequencing of two additional strains of the species [24].

To investigate the ability of *H. werneckii* to further adapt to high salinity, the reference strain of the species was transferred through a series of 100 batch subcultures in two separate populations in a liquid medium containing 4.3 M NaCl (25%; *w*/*v*) for a total of more than seven years and 800 generations. The observed changes at the phenotypic and genomic levels are described and interpreted in terms of growth under extreme salinity.

## 2. Materials and Methods

### 2.1. Experimental Evolution

A single colony of *Hortaea werneckii* EXF-2000 was inoculated into two Erlenmeyer flasks, each containing 200 mL of malt extract broth (MEB) with 4.3 M (25%; *w*/*v*) NaCl (MEB25: 2% malt extract (Merck, Darmstadt, Germany), 2% glucose (Kemika, Zagreb, Croatia), 0.1% bacto-peptone (Difco, Franklin Lakes, USA), 25% NaCl (Merck, Darmstadt, Germany); all *w*/*v*; pH 5.5; sterilised by autoclaving). This created two populations that were kept separate throughout the experiment. The cultures were incubated on a rotary shaker at 24 °C and 180 rpm until they grew well into the stationary phase. Then, 0.75 mL of this culture was transferred to the fresh MEB25 medium (a 1:266 dilution) and incubated in the same manner for each of the populations. This was repeated for 100 growth cycles, with an average interval of 28 days between subsequent reinoculations and a total duration of the experiment of 7 years and 8 months. Without accounting for cell death the culture thus went through 8.1 generations in each subcultivation and approximately 800 generations in total. For reinoculations 50 and 100, an aliquot of the culture (designated T50-1 or T50-2 and T100-1 or T100-2 for two independent replicates) was mixed with glycerol to a final concentration of 50% (*v*/*v*) and frozen at −80 °C for later analysis. A subsample of sample T100-1 was plated on a malt extract agar plate (MEA) containing 4.3 M NaCl, five individual colonies were selected, labelled T100-1A, T100-1B, T100-1C, T100-1D, and T100-1E, mixed with glycerol to a final concentration of 50% (*v*/*v*) and frozen at −80 °C for later analysis. The original strain and all evolved samples/strains were deposited to the Ex Culture Collection of Infrastructural Centre Mycosmo (MRIC UL) at the Department of Biology, Biotechnical Faculty, University of Ljubljana, Slovenia, under the EX numbers listed in Table 1.

### 2.2. Growth Measurements

*H. werneckii* progenitor (T0) and evolved strains (T50-1, T100-1; T50-2, T100-2) were grown in MEB supplemented with 4.3 M NaCl to mid-exponential growth phase and adjusted to a final optical density at 600 nm (OD_600_) of 0.5. These suspensions were then serially diluted 10-fold (1–10^2^ dilutions) in fresh MEB and spotted as 3 μL aliquots on MEA plates without osmolyte (control) or supplemented with 4.3 M NaCl, 3.0 M KCl, 3.0 M sorbitol, or 4.3 M glycerol. Experiments were performed in triplicate.

Growth was also examined in the liquid medium. The above overnight cultures were diluted to an OD_600_ of 0.05 and grown in 96-well microtiter plates in 200 µL of fresh MEB without osmolytes or with 4.3 M NaCl. Growth was monitored for up to 160 h by measuring OD_600_. All experiments were performed in triplicate, and replicate data were used to calculate arithmetic mean ± standard deviation (SD). A haemocytometer was used for the direct counting of cells in cultures grown in MEB containing 4.3 M NaCl at OD_600_ 0.5 and OD_600_ 1.0.

To assess the melanisation of the evolved *H. werneckii*, overnight cultures of the progenitor (T0) and the evolved strains (T50-1, T100-1; T50-2, T100-2) were adjusted to OD_600_ 0.5. These suspensions were then serially diluted 10-fold in MEB (1–10^2^ dilutions) and spotted in 3 μL aliquots on MEA plates without osmolyte or with sorbitol (1.0 M and 3.0 M), glycerol (2.2 M and 4.3 M), NaCl (1.8 M, 3 M, and 4.3 M), or KCl (1.8 M and 3 M). Melanisation was observed after 9 days, except for 1 M sorbitol (after 6 days) and 4.3 M NaCl (after 14 days). Experiments were performed in triplicate.

### 2.3. Quantification of Cell Morphology

*H. werneckii* progenitor (T0) and evolved strains (T50-1, T100-1; T50-2, T100-2) were grown in MEB without osmolytes or with 4.3 M NaCl to an OD_600_ of 0.5. Then, cells were mounted on microscope slides with an antifade Vectashield with DAPI (Vector Laboratories, Burlingame, USA). Images of two independent experiments were acquired using Axio Imager M2 (Carl Zeiss, Oberkochen, Germany) equipped with an HXP 120 C Illuminator and a high-resolution microscopy camera AxioCam Mrm (Carl Zeiss, Oberkochen, Germany). Blue (365 nm) and differential interference contrast images were acquired for each field. All discrete units—from free-floating single cells to chains of multiple cells separated by septa—are hereafter referred to as cell chains, with single cells treated as a special case of cell chains without septa. For each sample, the number of septa of 120 cell chains was counted. The length and width of 120 chains were measured using ZEN2012 software (Carl Zeiss, Oberkochen, Germany). The chain width was determined as the diameter of the widest cell in the chain measured perpendicular to the long-chain axis. Data were plotted using GraphPad Prism 5 software. Cell chain length and cell width were presented as box-and-whiskers plots, while the number of septa was presented as the percentage of chains without septa (single-cell units) or with 1, 2, 3, 4, or 5 septa (two- to six-cell units) per chain. The nonparametric Kruskal-Wallis test with pairwise comparisons and *post-hoc* Bonferroni corrections for multiple comparisons was used to compare cell chain length and cell width between samples (IBM SPSS Statistics version 19.0; IBM Corporation, Armonk, NY, USA). *p*-values of less than 0.05 indicated statistical significance.

To evaluate the effect of cell wall integrity on the cell morphology of evolved *H. werneckii*, the progenitor (T0) and the evolved strains (T50-1, T100-1; T50-2, T100-2) were grown in MEA without osmolytes or with 1.8 M, 3 M, or 4.3 M NaCl in the presence of the cell wall inhibitor caspofungin (final concentration in medium was 50 ng/mL). This concentration was chosen as the lowest concentration with observable effects (the tested concentrations were 0.5 ng/mL, 5 ng/mL, and 50 ng/mL). Microscopic images of two independent experiments were acquired as described above when cultures reached an OD_600_ of 0.5.

### 2.4. Genome Sequencing and Analysis

Whole genomes of samples T0 (original *H. werneckii* EXF-2000), T50-1, T100-1, and all five single-colony isolates of sample T100-1 were sequenced. Samples of cultures frozen at −80 °C were grown in 200 mL MEB25 on a rotary shaker at 24 °C and 180 rpm until the mid-exponential phase (OD_600_ approximately 0.8). The biomass was harvested by filtration and frozen in liquid nitrogen. The genomic DNA was isolated as described by Gostinčar et al. [23] and sequenced on the MiSeq platform using 300 bp paired-end reads. Sequencing data were deposited in GenBank under BioProject number PRJNA507731.

The haploid *H. werneckii* genome was computationally extracted from the diploid reference genome of *H. werneckii* EXF-2000 (GenBank GCA_002127715.1) using HaploMerger2, a pipeline for rebuilding assemblies of highly heterozygous genomes [25]. This haploid genome was used as the reference genome in all subsequent mappings. Quality control of the raw sequencing reads produced in this study, their trimming, mapping to the reference genome, sorting and deduplication was performed as described by Gostinčar et al. [23]. Genome coverage was calculated using bedtools 2.26.0 with the ‘genomecov’ option. Several additional modifications of this pipeline were tested for variant calling: reducing the minimum seed length of ‘bwa mem’ to 10, replacing ‘bwa mem’ with NextGenMap 0.5.5 [26] or Segemehl 0.3.4 [27], and replacing the T0 sequencing reads with simulated reads generated from the diploid *H. werneckii* reference genome using ART_Illumina 2.5.8 [28]. Variant calling was performed using Genome Analysis Toolkit 4.1.6.0 [29] according to “GATK Best Practices” but with the “hard filtering” option. The ploidy was set to 2.

Genome coverage data were analysed and visualised in R [30] using the packages ‘dplyr’ [31] and ‘ggplot2’ [32]. The per-nucleotide coverage data were plotted along the contigs of the haploid reference genome of *H. werneckii*. To identify the aneuploid regions, a rolling median of the sequencing depth in a 20 kbp window was calculated using the function ‘rollmedian()’ from the R package ‘zoo’ [33]. This rolling median was then normalised by subtracting the median sequencing depth of the whole genome and dividing the result by the median sequencing depth of the whole genome. This resulted in a dataset where, in a largely diploid genome, values around 0 were expected for diploid regions, values around 0.5 for triploid regions, and values around −0.5 for haploid regions. To objectively identify aneuploid regions, they were defined as those in which the sequencing depth values calculated above were either greater than 0.35 or less than −0.35. Due to substantial noise in the data, all genomic regions that met either of these criteria in continuous stretches of at least 1 kbp were merged if they were closer than 30 kbp, and the resulting list of putative aneuploid genomic regions was filtered to retain only those that spanned more than 50 kbp.

Annotation of *H. werneckii* genes with PANTHER IDs was performed using ‘pantherScore2.2.pl’ against the HMMs in PANTHER library version 15.0. Enrichment analysis was performed at http://www.pantherdb.org/ (accessed on 12 January 2020) using the binomial test and Bonferroni correction for multiple testing [34]. Annotation with Kyoto Encyclopaedia of Genes and Genomes (KEGG) orthology numbers was performed with the online server BlastKOALA using the database ‘family_eukaryotes’ and the annotated genes were assigned to KEGG pathways at http://www.genome.jp/kegg/tool/map_pathway.html (accessed on 12 January 2020) [35].

## 3. Results

A single strain of *H. werneckii* was inoculated into a high-salinity liquid medium (4.3 M NaCl) and subcultured one hundred times, with an average of 28 days and a minimum of 8.1 generations between each subculture. A selection of samples from both parallel populations at the middle (samples T50-1, T50-2) and end of the experiment (samples T100-1, T100-2) and five single-colony isolates from T100-1 were characterised by a series of phenotyping assays and whole-genome sequencing.

### 3.1. Phenotype of the Evolved Strains

**Figure 1:**
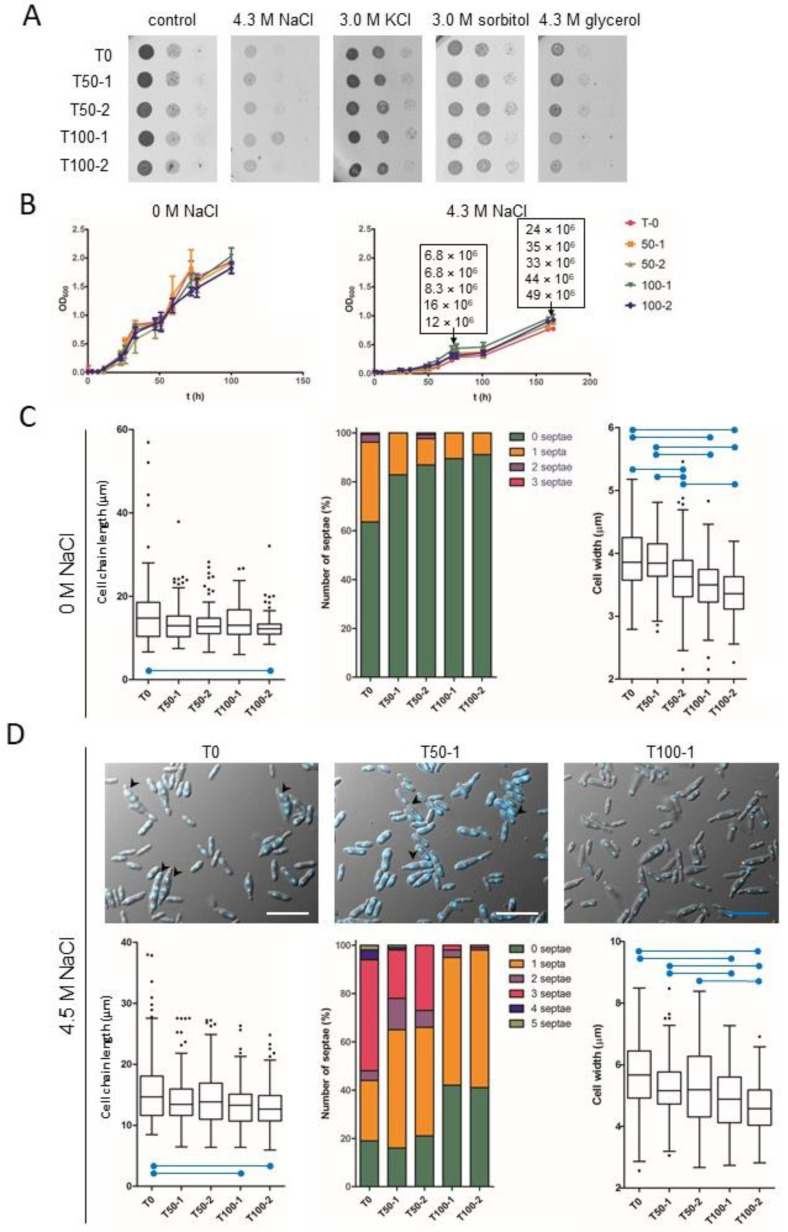
Growth rate and morphology of *Hortaea werneckii* during experimental evolution at high salinity. (**A**) *H. werneckii* progenitor (T0) and evolved strains (T50-1, T100-1; T50-2, T100-2); pre-cultures were diluted to OD_600_ 0.5 and two 10-fold serial dilutions were spotted onto MEA plates without osmolyte or with NaCl, KCl, sorbitol, or glycerol, as indicated. (**B**) Growth of *H. werneckii* progenitor and evolved strains in MEB without or supplemented with 4.3 M NaCl, as measured by OD_600_ over 160 h of incubation. Data are means ± standard deviation (SD) of triplicate experiments. Direct cell counts (number of cells per millilitre) of *H. werneckii* progenitor and evolved strains at OD_600_ 0.5 and 1.0 are listed within the rectangles in the graph (in the same order as in the graph legend). (**C**,**D**) Cell chain length, number of septa in individual cell chains and cell width of *H. werneckii* progenitor (T0) and selected evolved cultures (T50-1, T100-1) measured on 120 cell chains after growth to OD_600_ 0.5 in MEB without NaCl (panel C) or with 4.3 M NaCl (panel D), where significantly different (*p* ≤ 0.001) pairs of data are marked with blue horizontal lines. Outliers are marked with black dots. Measurements were made in two independent experiments that yielded similar data, with one representative experiment shown. (**D**) *H. werneckii* progenitor and evolved strains were grown in MEB with to OD_600_ 0.5. Microscopy images were then taken and processed as in panel C. Representative images of DAPI-stained *H. werneckii* cells of T0, T50-1, and T100-1 in MEB containing 4.3 M NaCl are shown in panel D. Black arrows mark multicellular chains with multiple septa. The length of the scale bars is 20 µm.

Growth of the progenitor (T0) and the evolved *H. werneckii* strains (T50-1, T100-1; T50-2, T100-2) was compared by spotting serial dilutions of cultures on solid media without osmolytes (control) or supplemented with 4.3 M NaCl, 3.0 M KCl, 3.0 M sorbitol, or 4.3 M glycerol (Figure 1A). Growth was also measured as OD_600_ of cultures in liquid media without osmolytes or with 4.3 M NaCl (Figure 1B). There were no obvious differences between strains, either in growth on solid media (Figure 1A) or in liquid media (Figure 1B). Because OD_600_ depends on several parameters, including the size and shape of the measured cells, and is therefore not necessarily proportional to cell density [36], we also estimated cell density by direct counting with a haemocytometer at two different time points (OD_600_ 0.5 and 1.0) during growth in MEB containing 4.3 M NaCl (Figure 1B, rectangular insets). Importantly, compared with the culture of the progenitor strain T0, the cell concentrations of cultures T100-1 and T100-2 were 2.4- and 1.8-fold higher at OD_600_ 0.5; they were 1.83- and 2.0-fold higher at OD_600_ 1.0. This indicated that the evolved strains differ in cell morphology from the progenitor strain. The changes in morphology were further examined by microscopy of liquid cultures in media without osmolytes or with 4.3 M NaCl, and the length of cell chains, cell width, and the number of septa per chain were analysed, treating single cells as a special case of cell chains without septa (Figure 1C,D).

When cultured in MEB in the absence of NaCl, significant differences in cell chain length were observed between samples, with the evolved strains having significantly shorter chains (Kruskal-Wallis test statistic = 17.908, *p* = 0.001), but only the difference between T100-2 and T-0 cultures was significant after Bonferroni correction for multiple comparisons (adjusted *p* < 0.001) (Figure 1C). The units of the evolved strains were mostly unicellular (about 90% of cell chains), whereas the culture of the progenitor strain contained about 40% chains with 1–3 septa (Figure 1C, middle). Significant differences were also found in cell width between samples (Kruskal-Wallis test statistic = 110.618, *p* < 0.001). Both T100-1 and T100-2 had significantly narrower cells compared to T-0 (both adjusted *p* < 0.001) and also compared to T50-1 (both adjusted *p* < 0.001). On the other hand, only T100-2 was significantly different from T50-2 (adjusted *p* < 0.001), while the difference between T100-1 and T50-2 did not withstand the correction for multiple comparisons (adjusted *p* = 0.503). T50-2 itself had significantly narrower cells compared to T-0 (adjusted *p* = 0.001) and compared to T50-1 (adjusted *p* < 0.001) (Figure 1C).

Significant differences in cell chain length were also observed between samples grown in MEB containing 4.3 M NaCl (Kruskal-Wallis test statistic = 19.886, *p* = 0.001). Under these hypersaline conditions, differences in chain length between T100-2 and T-0 and between T100-1 and T-0 remained significant after Bonferroni correction for multiple comparisons (adjusted *p* < 0.001 and *p* = 0.008, respectively) (Figure 1D). The final evolved cultures contained fewer septa in cell chains with approximately twice as many single cells in T100-1 and T100-2 than in T0, T50-1, and T50-2, which also contained 30–40% of chains with three or more cells—structures that were nearly absent in T100-1 and T100-2 (Figure 1D, top images, middle graph). Significant differences in cell width were also observed between samples at 4.3 M NaCl (Kruskal-Wallis test statistic = 65.147, *p* < 0.001). Both T100-2 and T100-1 had significantly narrower cells compared to T-0 (both adjusted *p* < 0.001) and compared to T50-1 (adjusted *p* < 0.001 and *p* = 0.045, respectively). On the other hand, only T100-2 was significantly different from T50-2 (adjusted *p* < 0.001), whereas the difference between T100-1 and T50-2 was not significant after correction for multiple comparisons (adjusted *p* = 0.095) (Figure 1D).

When comparing the cell morphology of strains grown in the absence or presence of 4.3 M NaCl, the cell chains of all strains were similar in length, but the cells were wider in the presence of NaCl (all *p* < 0.001, calculated using Mann-Whitney test) (Figure 1C,D).

**Figure 2:**
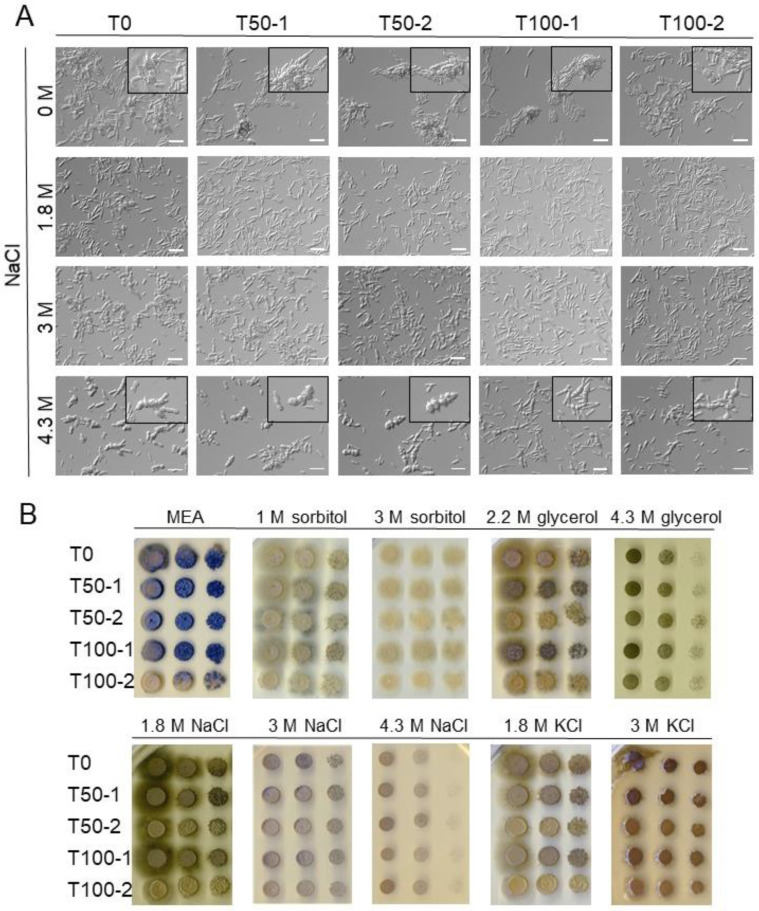
Morphology of *Hortaea werneckii* exposed to caspofungin and various osmolytes. (**A**) Progenitor (T0) *Hortaea werneckii* and evolved strains (T50-1, T100-1; T50-2, T100-2) were grown in MEB without osmolyte or with 1.8 M, 3 M, or 4.3 M NaCl in the presence of 50 ng/mL of the cell wall synthesis inhibitor caspofungin. Microscopy images were taken when the cultures reached an OD_600_ of 0.5. Two independent experiments yielded similar data, and the representative images of one experiment are shown. The length of the scale bars is 20 µm. Inlays show representative examples of casponfungin-caused changed morphology. (**B**) Melanisation of *H. werneckii* progenitor and evolved strains grown in the presence of different solutes. Pre-cultures diluted to OD_600_ 0.5 were spotted at 10-fold serial dilutions on MEA plates without osmolyte or with sorbitol, glycerol, NaCl, or KCl as indicated. Images were taken after 9 days incubation, except for 1 M sorbitol (taken after 6 days) and 4.3 M NaCl (taken after 14 days). The experiment was performed in triplicate and the representative images are shown.

One reason for the narrower cells with a smaller number of septa in the more evolved strains could be due to changes in the cell wall. To test this, the progenitor (T0) and the evolved strains (T50-1, T100-1; T50-2, T100-2) were grown in MEB without osmolyte or with 1.8 M, 3 M, or 4.3 M NaCl, in the presence of the cell wall synthesis inhibitor caspofungin. Caspofungin is a lipopeptide antifungal agent that inhibits the synthesis of the fungal cell wall component β-(1,3)-D-glucan, thereby disrupting the cell wall integrity [37]. Microscopy images of a representative experiment are shown in Figure 2A. Due to the unusual morphology of the cell chains and the presence of larger cell aggregates in the cultures, it was not possible to measure growth with OD_600_ and quantify cell morphology. However, in the presence of caspofungin in a non-saline medium, fewer chains with atypical morphology were observed in the more evolved strains (T100-1, T100-2) compared to the progenitor strain T0 (Figure 2A, first row). In the presence of caspofungin and 4.3 M NaCl, cell chains in all strains became visibly broader and contained more septa (Figure 2A, rows 2–4).

Because melanin is an important component of *H. werneckii* cell walls, we also looked for qualitative differences in the melanisation of colonies on MEA plates supplemented with different osmolytes (Figure 2B). There was a marked difference in melanisation between the evolved strains of the two experimental populations. In the first population, colonies of evolved strains (T50-1, T100-1) were more melanised than the progenitor strain (T0), which was best observed on MEA supplemented with 2.2 M glycerol, 1.8 M NaCl, or 1.8 M KCl. The opposite was true for the second population: strains T50-2 and T100-2, grown on the same plates, were less melanised compared to T0, with T100-2 showing an almost complete loss of melanisation under the conditions tested.

Filamentous growth decreased in both T100-1 and T100-2 on MEA without added osmolytes (Figure 2B). Nevertheless, filamentous growth was observed in all strains when grown on MEA with sorbitol, 2.2 M glycerol, 1.8 M NaCl, or 1.8 M KCl.

### 3.2. Genomic Changes in the Evolved Strains

The median sequencing depth calculated per haploid *H. werneckii* genome was 36 (T0), 145 (T50-1), 131 (T100-1), 113 (T100-1A), 102 (T100-1B), 85 (T100-1C), 98 (T100-1D) and 62 (T100-1E). Variant calling in the sequenced genomes of the evolved strains by comparing their sequences with the haploid reference sequence of *H. werneckii* was unsuccessful due to the poor performance of the variant calling pipeline even after several optimisation attempts. The failure could have been caused by the high heterozygosity of the strains, an unusually high sequencing bias against one or more alleles in some multiallelic loci, or some other reason that could not be identified. The number of heterozygous single nucleotide polymorphism (SNP) loci identified in the sequenced samples ranged from 1.10 to 1.25 million per genome, which was not unexpected since highly heterozygous diploid genomes were mapped to a haploid reference. However, after removing all variant loci found in sample T0, the other genomes still contained approximately 80,000 heterozygous SNP loci (approximately 0.4% of a diploid *H. werneckii* genome). Upon closer inspection of a single contig, the majority of these loci were already confirmed as heterozygous in the reference genome but were not detected as such in the T0 genome. This was due to short, randomly distributed sections of the reference genome covered by reads from only one haploid subgenome, giving the false impression that these loci were homozygous in T0 and had acquired heterozygous SNPs in the evolved strains. While this looked like the result of sequencing bias, the problem persisted when the T0 reads were replaced with mock data obtained by simulating reads from the diploid reference genome, suggesting a problem with mapping rather than sequencing. Using a different mapping algorithm and changing the mapping parameters did not improve the result sufficiently to allow reliable variant calling.

**Figure 3:**
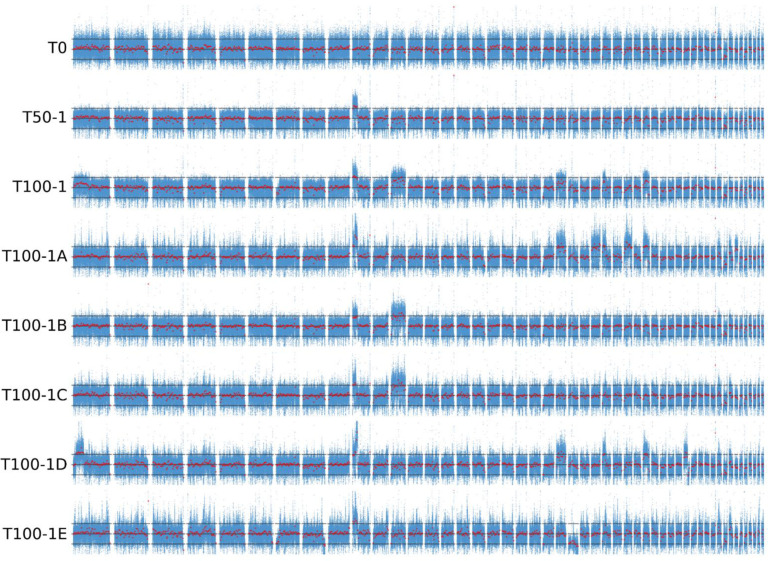
Sequencing coverage of the progenitor and evolved strains of *Hortaea werneckii*. Whole-genome sequencing reads were mapped to a haploid *H. werneckii* reference genome. The per-nucleotide sequencing depth was normalised by the median sequencing depth of the strain by subtracting the median from the per-nucleotide depth and dividing the result by the median. For the 50 largest contigs of the haploid reference genome, arithmetic mean coverage in 50 bp bins is shown in blue, overlayed by the median coverage in 30 kbp bins (red). Horizontal lines mark the values 0 (approximately expected coverage for diploid regions in a majority diploid genome), −0.5 (approximately expected coverage for haploid regions), and 0.5 (approximately expected coverage for triploid regions).

Mapping the sequencing reads to the haploid reference genome and calculating the depth per nucleotide showed that some contigs had much higher, and in some cases lower, sequencing depth than the rest of the genome (Figure 3). In these regions, coverage was approximately 50% higher or lower than the median coverage of the whole genome, which was interpreted as evidence that these regions were triploid or haploid, respectively. Consistent with this interpretation, in these regions the ratio of reads representing different alleles at heterozygous biallelic loci deviated from the 1:1 ratio expected for diploids. Apart from the single haploid contig of T100-1E, no major regions of loss of heterozygosity were observed (Figure S1). Similar signs of aneuploidisation were observed in some wild isolates of *H. werneckii* sequenced in a previous study [23] and analysed here (Figure S2). During the experimental evolution at high salinity, the number of aneuploidies appeared to increase with the duration of the experiment. While no large aneuploid regions were observed in the original strain T0, at least one was observed in culture T50-1 and at least two in T100-1 (Figure S3). More importantly, the single-colony isolates of T100-1 (T100-1A to T100-1E) differed in the number and location of aneuploid regions, with only T100-1B and T100-1C sharing the same pattern of two triploid regions. T100-1D had five triploid regions, T100-1A had eight, and T100-1E had only one, but was also the only strain with a large haploid region. Of 11 wild *H. werneckii* isolates (Figure S3), seven had no large aneuploid regions, two strains had one and three triploid regions (J and K, both isolated from spider webs in Atacama, Chile), and the last two strains, clinical isolates from Brazil (E) and Italy (F), stood out with 16 triploid regions in strain E and three triploid and four haploid regions in strain F. Since the reference genome of *H. werneckii* was not assembled to the chromosome level, it is possible that some of these aneuploid regions actually belong to the same chromosome and represent parts of a single larger aneuploidy, which means that the actual number of aneuploidisation events counted above may be overestimated.

**Figure 4:**
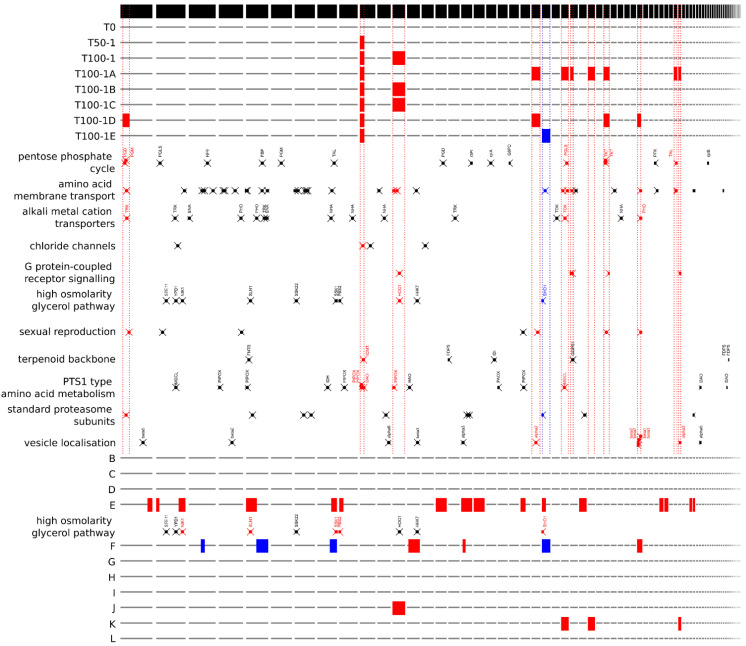
Position of aneuploid regions and selected gene groups in different strains of *Hortaea werneckii*. The location of the putative triploid (red) and haploid (blue) genomic regions is marked in relation to the contigs of the reference haploid genome, from left to right, from the longest to the shortest (black stripe on top). Evolved strains (T0 to T100-1E) and wild strains (B to L) of *H. werneckii* are shown in rows. Between them, the locations of selected genes are arranged in functional groups. Gene locations are marked with crosses. Genes were identified by PANTHER IDs (amino acid membrane transport, chloride channels, sexual reproduction, vesicle localisation), by KEGG orthology numbers (pentose phosphate cycle, G protein-coupled receptor signalling, terpenoid backbone synthesis, standard proteasome subunits, PTS1 type amino acid metabolism) or identified by manual searches for previously identified genes (alkali metal cation transporters, high osmolarity glycerol pathway).

Some functional gene groups identified by PANTHER annotation were found to be significantly overrepresented in aneuploid regions of the evolved strains (Figure 4). Triploid regions present in at least one of the evolved strains, which comprised 984 of the total 7807 genes annotated in the haploid reference genome of *H. werneckii*, contained five of five genes assigned to the GO-Slim biological process ‘G protein-coupled receptor signalling pathway’; 11 of a total of 37 genes assigned to the process ‘amino acid transmembrane transport’; and four of seven genes assigned to the category ‘sexual reproduction’. The tenth-largest contig, which was partially triploid in all evolved strains, contained 1 of 4 genes encoding proteins with putative chloride transmembrane transporter activity. The only large haploid region in any of the strains generated by experimental evolution contained one of a total of two genes in the ‘regulation of exocytosis’ and ‘oxidative demethylation’ categories.

The triploid regions of clinical isolate E contained six of ten genes classified in the ‘vesicle localization’ process and the triploid regions of clinical isolate F were most enriched in the ‘spliceosomal complex assembly’ and ‘homologous recombination’ categories, whereas its haploid regions were most enriched in the ‘telomere capping’ category.

**Figure 5:**
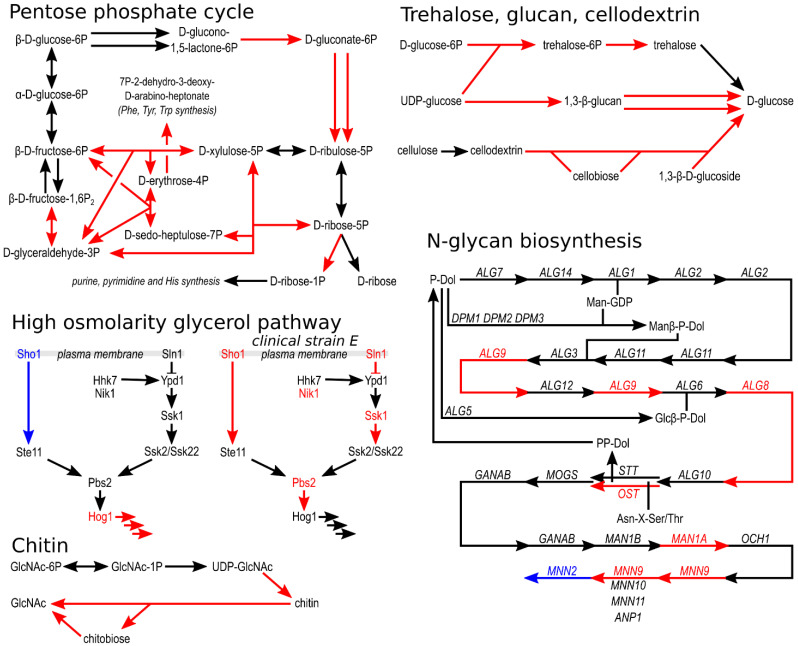
Schematic representation of selected metabolic pathways in *Hortaea werneckii*. Predicted proteins of the haploid reference *H. werneckii* genome were assigned to KEGG pathways. These were redrawn to show selected pathways. Arrows mark enzymatic reactions with putative enzymes identified in *H. werneckii*. The presumed ploidy of the genomic region with the gene for a particular enzyme is indicated by the colour of the arrow and the name of the gene/protein: diploid in black, triploid in red, haploid in blue. If a particular enzyme is coded by genes in more than one locus, at least one of these loci had to be in aneuploid regions of at least one of the evolved strains to be marked with red or blue.

The assignment of *H. werneckii* genes to KEGG pathways revealed that for some pathways a large proportion of assigned genes occurred in aneuploid regions (Figure 5). Among the most notable examples is the pentose phosphate cycle, where genes for seven of fifteen enzymes found were in triploid regions in at least one of the evolved strains. Several genes in triploid regions were also found in the synthesis and degradation of β-glucan and chitin, the degradation of cellodextrin, and the synthesis of N-glycans and trehalose (Figure 5).

A manual search for groups of previously identified *H. werneckii* genes revealed that of 15 alkali metal cation transporter genes, only three were found in triploid regions, one each of the potassium transporters TRK and TOK and one PHO Na^+^/P_i_ importer (Figure 4). Of the nine genes encoding components of the high-osmolarity glycerol (HOG) signalling pathway, only HOG1, encoding the central mitogen-activated protein kinase of the pathway, was located in the triploid region of two evolved strains (T100-1B and T100-1C, and in wild isolate J), and SHO1, the gene encoding the transmembrane osmosensor, was located in the haploid region of T100-1E (Figure 4 and Figure 5). In clinical isolate E, five of nine components of HOG were located in triploid regions, whereas in clinical isolate F one gene was located in a triploid region and two genes were located in haploid regions. 

## 4. Discussion

Even after seven years of growth at 4.3 M NaCl, the growth rate of *Hortaea werneckii* did not change. Although the experimental conditions were well above the salinity optimum (0.8–1.7 M NaCl) of the species [20], this seemed to indicate an already high fitness of the wild strain with little room for further improvement—a very different outcome than observed in the experimental evolution of the halotolerance of salt-sensitive species. For example, *S. cerevisiae* exposed to high NaCl concentrations for many generations evolved a faster growth rate under high-salt conditions; evolution of *E. coli* under high-sorbitol stress resulted in improved growth at high osmolarity and reduced growth at low osmolarity [38]. Perhaps more surprisingly, the ability of *H. werneckii* to grow in the absence of salt—an ability that seems to be rarely used in the wild [19]—did not diminish even after years of no exposure to mesophilic conditions.

However, on closer inspection, changes at both phenotypic and genomic levels were also observed in evolved strains of *H. werneckii*. The most obvious adjustment in cell morphology was a significant decrease in cell width and a moderate decrease in the length of chains of cells, a morphology typical for *H. werneckii*. The latter reflects the decrease in the number of cells per chain in the evolved strains. While much of the progenitor culture consisted of multicellular chains, experimental evolution led to an increase in the number of unicellular units.

Due to an unacceptably high number of false-positive short variants generated by the variant calling pipeline, genomic analysis focused on ploidy changes of evolved *H. werneckii* genomes. The relative role of small mutations compared to large-scale aneuploidies was therefore not investigated. There was a striking increase in the number of aneuploidies in the genome of the evolved strains. As discussed below, the aneuploid regions of the evolved strains were significantly enriched in some functional gene groups. If the cellular functions of these genes were advantageous or disadvantageous under conditions of experimental evolution, and this effect was adjusted by changes in gene dosage, this could explain why at least one aneuploidy persisted over many generations.

### 4.1. Morphology

Cell size may play an important role in cell fitness, affecting susceptibility to predators [39] and sensitivity to antimicrobials [40], among other factors. The cells of *H. werneckii* became significantly narrower during the first 50 subcultures at high salinity in one experimental population and between subcultures 50 and 100 in the other population. The reduced width of cells in the evolved *H. werneckii* contrasts with the observations of the Lenski experiment, in which the size of *E. coli* cells increased concomitantly with an increase in fitness [41]. However, the mutations associated with this change in *E. coli* were associated with increased sensitivity to osmotic stress [42]. Similarly, *E. coli* cells selected for increased UV tolerance increased in size and resistance to desiccation and starvation but became much more salt-sensitive [43]. However, experiments with *S. cerevisiae* showed that the relationship between halotolerance and cell size can vary—experimental evolution at high salinity resulted in an increase in yeast cell size, explained by increased ploidy of the evolved strains [14]. In addition to adaptation to high salinity, the reduced width of *H. werneckii* cells could also be an indirect consequence of changes in solute management of the evolved strains. Na^+^, K^+^, and Cl^−^ are major determinants of cell osmotic stability, but cellular ion fluxes are also closely related to cell size [44]. With this in mind, it is perhaps interesting that genes for two potassium transporters and one Na^+^/P_i_ transporter were found in triploid regions of evolved *H. werneckii* strains and, perhaps more importantly, that a gene for one of the putative chloride transmembrane transporters was found in the region that was triploid in all evolved strains. The cellular management of chloride in halophilic and halotolerant organisms has attracted little research attention compared to the much better-understood management of sodium and potassium [45], leaving room for further studies.

Aneuploid regions could also be related to other observed changes in the morphology of evolved *H. werneckii* strains. The altered organisation of cell chains could be caused by changes in cell wall composition or organisation—which would also be consistent with the decreased sensitivity of the evolved strains to caspofungin, an inhibitor of β-(1,3)-D-glucan synthesis. For example, some of the genes in triploid regions encode proteins involved in the synthesis and/or degradation of β-glucan, chitin, cellodextrin, and N-glycans (Figure 4 and Figure 5). The cell wall determines the shape and shape change of fungal cells [46]. It must also provide mechanical strength, and to do so under different conditions, it must dynamically adapt by changing its composition, which in fungi generally consists of branched β-(1,3)-glucan and chitin [47], along with several other compounds, including highly mannosylated glycoproteins, with N-glycans being the main form of mannoprotein modification [46]. Yeast cells utilise the cell wall integrity signalling pathway (CWI), the high-osmolarity glycerol pathway (HOG), and the calcineurin signalling pathway to trigger a rapid structural realignment of the cell wall composed of interlinked β-glucan and chitin, ensure its elasticity, and increase survival during osmotic shock [48,49].

Cell wall adaptation has been shown by several analyses of fungi from extreme environments. In the halophilic *Eurotium rubrum*, the salt-responsive gene set was enriched in genes involved in beta-glucan biosynthetic processes and chitin binding [50]. In the halotolerant *Aspergillus sydowii*, high salinity triggered an increase in the expression of endochitinase and chitotriosidase, as well as genes involved in the synthesis and modification of β-glucans [51]. In the halophilic basidiomycete *Wallemia ichthyophaga*, which can only grow in hypersaline media, the unusually thick cell wall further thickens at high salinity, which also up-regulates the expression of an exo-1,3-β-glucanase [52], of two endoglucanases and a glucan 1,3-β-glucosidase genes, and down-regulates the expression of genes for seven other endoglucanases, two expansin-like proteins, an endo-1,3(4)-β-glucanase, and two 1,3-β-glucan synthases [53]. Transcriptome analysis of *H. werneckii* itself showed that the putative genes for 1,3-β-glucanosyltransferases were among the most upregulated genes at high salinity [54].

An increase in unicellular units in the evolved *H. werneckii* strains was unexpected. The clustering of cells into densely packed meristematic clumps observed in numerous black fungi and *W.* *ichthyophaga* is thought to serve as protection against stress [55,56]. However, the aggregation of *H. werneckii* cells typically takes the form of linear chains formed by fission and budding [57], rather than meristematic clumps formed by isodiametric growth. The decrease in size of these cell chains during experimental evolution suggests that at least this form of aggregation is not an adaptation to growth at constant high salinity.

Finally, a characteristic feature of *H. werneckii* is the accumulation of melanin in the cell walls. This accumulation is thought to play an important role in the halotolerance of the species [58,59]. However, the different response of the two experimental populations of *H. werneckii* during the evolution at high salinity—an increase in melanisation in one population and a decrease in the other—suggests that the adaptive value of melanisation at constant high salinity is either lower than generally assumed or that it is linked to other adaptations in a way that is currently unknown.

### 4.2. Aneuploidy

Ploidy shifts are not necessarily adaptive [60], but several authors have reported ploidy changes accelerated by salt stress in fungi [61]. The experimental evolution of *S. cerevisiae* under continuous NaCl stress resulted in modest changes in the expression of some genes and a substantial increase in genome size [14], while adaptation of *S. cerevisiae* to low temperatures, sorbitol, and NaCl resulted in a strain with improved halotolerance and reduced genome size [15].

The first triploid region identified in the experimental evolution of *H. werneckii* was already clearly visible in sample T50-1. This aneuploidy was inherited by all sequenced genomes from the sample T100-1, where several additional aneuploidies appeared (Figure 3). Aneuploidies can fundamentally alter the functioning of the cell and usually have a much greater impact than polyploidy [62]. Both polyploidy and aneuploidy are increasingly recognised as widespread transient adaptations of fungi to novel conditions (see Naranjo–Ortiz and Gabaldón [63], Tsai and Nelliat [64]). The effects of aneuploidy range from simple increases in gene expression due to increased gene dosage, to cascades of changes caused by altered expression of global regulators or impaired assembly of macromolecular complexes due to changes in stoichiometry [65]. While changes in ploidy can also disrupt sexual reproduction [60], this should not affect the strictly clonal *H. werneckii* [23]. Interestingly, while in *S. cerevisiae* experimental evolution of diploid strains caused frequent loss-of-heterozygosity [9], this did not occur on a larger scale in *H. werneckii* (Figure S1), but cannot be fully ruled out at smaller scales, where it would remain hidden in the noise of the unreliable identification of heterozygous alleles.

Even if aneuploidies in the evolved strains of *H. werneckii* are indeed adaptive, they are not necessarily part of the adaptation to high salinity but could also increase fitness under the artificial conditions of the experiment, for example by helping cells to efficiently use the resources available in the medium. The high availability of simple sugars and an abundance of other nutrients are far from the typical conditions encountered by *H. werneckii* in its natural habitat [19,66]. Such adaptation to laboratory conditions has been noted in numerous other studies of experimental evolution. For example, in the long-term experimental evolution of *E. coli*, cells achieved higher maximum growth rates on glucose and one strain even acquired the ability to aerobically utilise citrate [67].

Whatever the reason for the occurrence, spread, and persistence of aneuploidies in *H. werneckii*, the phenomenon is not restricted to experimental evolution. Our analysis revealed that aneuploidies were detected in four of eleven sequenced wild isolates of *H. werneckii* (Figures S2 and S3): two of four strains from the seashore cave in Atacama Desert and two of three clinical isolates. Notably, the clinical isolates contained multiple aneuploidies. For a rare opportunist like *H. werneckii*, the human body is a novel and probably very stressful environment [68]. The same is true for the hypersaline laboratory medium. While aneuploidisation is generally disruptive, it can be beneficial in adverse or rapidly changing environments, facilitating survival and further adaptation [63,69,70]. It is generally considered a temporary solution, but in some cases appears to be stable over longer periods of time [71]. In *S. cerevisiae* subjected to abrupt heat stress, chromosomal duplications were observed repeatedly, but were subsequently supplanted by solutions obtained at the level of individual genes; and if the stress was applied gradually, they did not occur at all [72]. The polysomy of chromosome III has been reported to be the major genomic feature of highly ethanol-tolerant *S. cerevisiae* strains [73]. Peter et al. observed aneuploidies in over 20% of over 1000 sequenced *S. cerevisiae* strains [74]. Aneuploidies are commonly found in clinical isolates of *Candida albicans*, sometimes increasing antimycotic resistance. Aneuploidies regularly occur in systemic fungal pathogens during infection [75,76]. Such adaptation is particularly useful for microorganisms but is also observed in multicellular organisms. It has been studied in plants [65] and is an important feature of most cancers, where aneuploidy correlates with poor prognosis, increased metastatic potential, and drug resistance [77].

In wild diploid *H. werneckii* strains, aneuploidy could be the result of genome reconfiguration, after these strains presumably arose by hybridisation between highly heterozygous haploid strains. While exposure to a novel environment and unusual hybrid genomes could both be associated with the emergence of aneuploidy, the lack of ploidy variation in the majority of diploid genomes [23], including clinical isolate H, suggests that some or all of these speculations may be oversimplified and that further research is needed to thoroughly test each of them.

Different aneuploidies in individual single-colony isolates (with only two out of five sharing the same pattern) showed that after 100 subcultures the culture was in fact a mixture of (at least four and probably more) different lineages with different mutations, which could coexist at least in the short-term. Only some of these lineages represented a sufficient proportion of the population to cause visible changes in the per-nucleotide sequencing depths of the entire T100-1 pool (Figure 3). No such coexisting lineages evolved in the long-term evolution of *S. cerevisiae* [9] but they were observed in several other studies of experimental evolution. Different lineages of *E. coli* were found to evolve and in some cases coexist for many generations in the Lenski experiment, an observation explained by cross-feeding or specialisation for different phases of an ageing culture [3]. Other authors have shown that even very homogeneous environments can harbour microniches that support the growth of different aneuploids (reviewed in Naranjo–Ortiz and Gabaldón [63]).

### 4.3. Pentose Phosphate Cycle

One of the KEGG metabolic pathways where gene dosage was most elevated in the evolved *H. werneckii* strains was the pentose phosphate cycle. From here, one of the pathways leads to the formation of D-ribose-1-phosphate (and from there to purine, pyrimidine and histidine synthesis) using an enzyme classified as phosphopentomutase/phosphoglucomutase, which is not obviously related to adaptation to increased salinity, but phospho-sugar mutases have been implicated in adaptation to salt stress before. Phospghoglucomutase and phosphomannomutase genes from the halotolerant yeast *Rhodotorula mucilaginosa* increased NaCl and LiCl tolerance of *S. cerevisiae* when heterologously overexpressed [78]. Phosphoglucomutase is known to be extremely sensitive to lithium ions because it competes with magnesium, a cofactor of both phosphoglucomutases and phosphopentomutases [79]. Another gene from *Rhodotorula mucilaginosa* that increased halotolerance of *S. cerevisiae* was a 3-deoxy-D-arabinoheptulosonate-7-phosphate synthase that leads from the pentose phosphate pathway to phenylalanine, tyrosine, and tryptophan biosynthesis, thereby regulating the amount of substrate entering the pathway—the gene for this enzyme was also found in triploid regions of evolved *H. werneckii* strains. Finally, transketolase and 6-phosphogluconate dehydrogenase, two other enzymes of the pentose phosphate pathway whose genes were found in triploid regions of the evolved *H. werneckii* strains, were previously found to be strongly induced by high salinity in both *H. werneckii* and *S. cerevisiae* [14,80]. Evolutionary engineering of *S. cerevisiae* for halotolerance increased the expression of transketolase and 6-phosphogluconolactonase genes [16].

### 4.4. Signalling Pathways

In order to respond to environmental conditions, these must first be detected and the information conveyed further. The high osmolarity glycerol (HOG) mitogen-activated protein kinase (MAPK) pathway is the main signalling pathway for the sensing of osmolarity changes in *S. cerevisiae*. It has been extensively studied in *H. werneckii* [81,82,83,84]. The HOG signalling pathway is coordinated with the cell wall integrity (CWI) signalling pathway [85] and thus may be associated with changes in the cell wall of evolved *H. werneckii* strains. The presence of the major MAPK gene of the pathway, HOG1, in the triploid regions of two genomes and the presence of the osmosensor gene SHO1 in a haploid region of one genome could lead to altered osmosensing and altered expression of the many HOG-regulated genes. The localisation of five out of the nine HOG-pathway genes in triploid regions of clinical isolate E (but not HOG1 itself) might be related to host adaptation of this strain. In fungal pathogens, the HOG pathway plays several roles important for virulence in addition to its usual role in coordinating the osmotic stress response [86]. This conclusion is seemingly contradicted by the fact that two of the five genes with increased dosage in isolate E had a decreased dosage (location in haploid regions) in the clinical isolate F—or this could simply indicate different ways of adapting the HOG pathway for pathogenesis.

Other pathways may play a role in *H. werneckii* haloadaptation, as indicated by the significant enrichment of G-protein-coupled receptor-binding proteins in the triploid regions of strains that evolved at high salinity. Because fungal G-protein-coupled receptors sense a variety of signals, from hormones, proteins and nutrients, to ions, hydrophobic surfaces, and light [87], the potential role of the observed enrichment is unclear.

### 4.5. Compatible Solutes, Ion Transporters, Virulence

Triploid regions of evolved *H. werneckii* strains contained two enzymes for the synthesis of trehalose. While glycerol has been identified as the major compatible solute of *H. werneckii* [88], trehalose has a well-known stress-protective function in both eukaryotes and prokaryotes, protecting not only against osmotic stress but also against desiccation, oxidation, and temperature fluctuations [89]. It has been reported that the evolved *S. cerevisiae* strain with increased halotolerance contained significantly increased amounts of trehalose and glycogen [16]. The overrepresentation of genes involved in ‘amino acid transmembrane transport’ could allow better utilisation of resources provided in the medium or accumulation of amino acids and their derivatives as compatible solutes.

The very low presence of genes encoding alkali metal cation transporters in the aneuploid regions of evolved *H. werneckii* strains is perhaps unexpected, considering that these transporters play a critical role in maintaining the proper intracellular balance of Na^+^, K^+^, and other ions. Of course, the activity of these transporters can be regulated at other levels and does not require adjustment due to the already high halotolerance of *H. werneckii*. Furthermore, alkali metal cation transporters may not be a particularly good indicator of halotolerance. While *H. werneckii* contains many copies of genes encoding Na^+^, K^+^ membrane transporters [21], this is also true for several other phylogenetically related fungi, some of which are not particularly halotolerant [90]. Moreover, the number of genes for these transporters in the most halophilic fungus known, *W. ichthyophaga,* is completely unremarkable, and even their expression is not responsive to high salinity [53].

In clinical isolates of *H. werneckii* the enrichment of genes in the ‘vesicle localization’ category may be related to modulation of the switching between yeast and filamentous growth, a process important for fungal pathogenesis that is associated with vesicle transport [91]. Enrichment of genes in the ‘spliceosomal complex assembly’ category may be related to the importance of splicing regulation during fungal pathogenesis, discussed by Sesma [92], and enrichment of genes in the ‘homologous recombination’ category may be important for genome plasticity, which is often observed during infection [93].

## 5. Conclusions

The experimental evolution of an already extremely halotolerant fungus at high salinity might not be expected to lead to further adaptation. Indeed, the growth rate of the evolved *H. werneckii* strains appeared to remain unchanged even after more than seven years of growth at 4.3 M NaCl. However, several changes were observed during evolution, both at the morphological and genomic levels.

A significant reduction in cell width of the evolved strains, a reduced number of cells arranged in short chains typical of *H. werneckii*, reduced sensitivity to the cell wall synthesis inhibitor caspofungin, and an altered melanisation of cell walls suggest an important role of cell morphology for growth at constant high salinity. Several genes encoding cell wall-modifying enzymes were found in genomic regions that became triploid in at least one of the evolved strains. Aneuploidies, previously described as a hallmark of rapid adaptation to severe stress in a variety of cells, from unicellular fungi to cancer cells, also appear to be beneficial in the adaptation of *H. werneckii* to high salinity *in vitro*. Aneuploidies have also been detected in wild strains, including two of three sequenced clinical isolates of the species. Explaining the effects of aneuploidies is not straightforward because of the large number of genes involved. However, some patterns have been identified that are consistent with observed phenotype changes or results of previous studies of adaptation to high salinity. These patterns may be useful in directing future research on the exceptional halotolerance of *H. werneckii*.

Whole-genome sequencing of additional strains, particularly from an independently evolved second experimental population, could confirm or refute some of the explanations proposed above. If advances in methodology can overcome the intractability of *H. werneckii* to genetic manipulation, targeted genetic experiments should provide additional explanations for fungal halotolerance. For such experiments, this study identifies at least three potential foci for further exploration of fungal halotolerance: the cell wall, the pentose phosphate cycle, and components of signal transduction pathways in addition to the canonical osmotic stress signalling pathways.

## Figures and Tables

**Table 1:** Strains of *Hortaea werneckii* compared in this study.

Name in This Study	Ex Culture Collection Strain Number	Genome Sequence or Sequencing Reads (GenBank)	Notes
T0	original strain: EXF-2000; stored again in this study as: EXF-14105	reference genome: GCA_002127715.1 *; this study: SRR15276557	progenitor strain used in the experimental evolution, isolated from hypersaline water of Sečovlje saltpans, Slovenia; reference genomic strain
T50-1	EXF-14106	this study: SRR15276556	strain (pool) after 50 subcultures at high salinity; parallel experiment 1
T50-2	EXF-14107	NA	strain (pool) after 50 subcultures at high salinity; parallel experiment 2
T100-1	EXF-14108	this study: SRR15276555	strain (pool) after 100 subcultures at high salinity; parallel experiment 1
T100-2	EXF-14109	NA	strain (pool) after 100 subcultures at high salinity; parallel experiment 2
T100-1A	EXF-14100	this study: SRR15276554	single-colony isolate from T100-1
T100-1B	EXF-14101	this study: SRR15276553	single-colony isolate from T100-1
T100-1C	EXF-14102	this study: SRR15276552	single-colony isolate from T100-1
T100-1D	EXF-14103	this study: SRR15276551	single-colony isolate from T100-1
T100-1E	EXF-14104	this study: SRR15276550	single-colony isolate from T100-1
B	EXF-120	GCA_003704685.1 **	wild isolate from hypersaline water, Santa Pola saltpans, Spain
C	EXF-562	GCA_003704675.1 **	wild isolate from soil on the sea coast, Namibia
D	EXF-2788	GCA_003704645.1 **	wild isolate from hypersaline water, Sečovlje saltpans, Slovenia
E	EXF-171	GCA_003704615.1 **	wild isolate from human Keratomycosis, Brasil
F	EXF-2682	GCA_003704585.1 **	wild isolate from human Trichomycosis nigra, Italy
G	EXF-10513	GCA_003704595.1 **	wild isolate from deep sea water, Italy
H	EXF-151	GCA_003704575.1 **	wild isolate from human Tinea nigra, Portugal
I	EXF-6651	GCA_003704385.1 **	wild isolate from a spider web, seashore cave in Atacama Desert, Chile
J	EXF-6669	GCA_003704355.1 **	wild isolate from a spider web, seashore cave in Atacama Desert, Chile
K	EXF-6654	GCA_003704375.1 **	wild isolate from a spider web, seashore cave in Atacama Desert, Chile
L	EXF-6656	GCA_003704345.1 **	wild isolate from a seashore cave rock wall, Atacama Desert, Chile

* Sinha et al. [22]. ** Gostinčar et al. [23].

## Data Availability

Sequencing data are available in GenBank under BioProject number PRJNA507731.

## Supplementary Materials

The following are available online at https://www.mdpi.com/article/10.3390/jof7090723/s1, Figure S1: Allelic ratio of heterozygous biallelic loci in the progenitor and the evolved strains of *Hortaea werneckii*, Figure S2: Sequencing coverage of wild isolates of *Hortaea werneckii*, Figure S3: Sequencing coverage and aneuploid regions of *Hortaea werneckii* genomes.
