# Supplementary material for: Seven Years at High Salinity—Experimental Evolution of the Extremely Halotolerant Black Yeast Hortaea werneckii
Source: J Fungi (Basel). 2021 Sep 4;7(9):723. doi: 10.3390/jof7090723 (PMC8468603; doi:10.3390/jof7090723)
Supplement: Supplementary file 1 [file jof-07-00723-s001.zip › jof-1360571-supplementary.pdf]

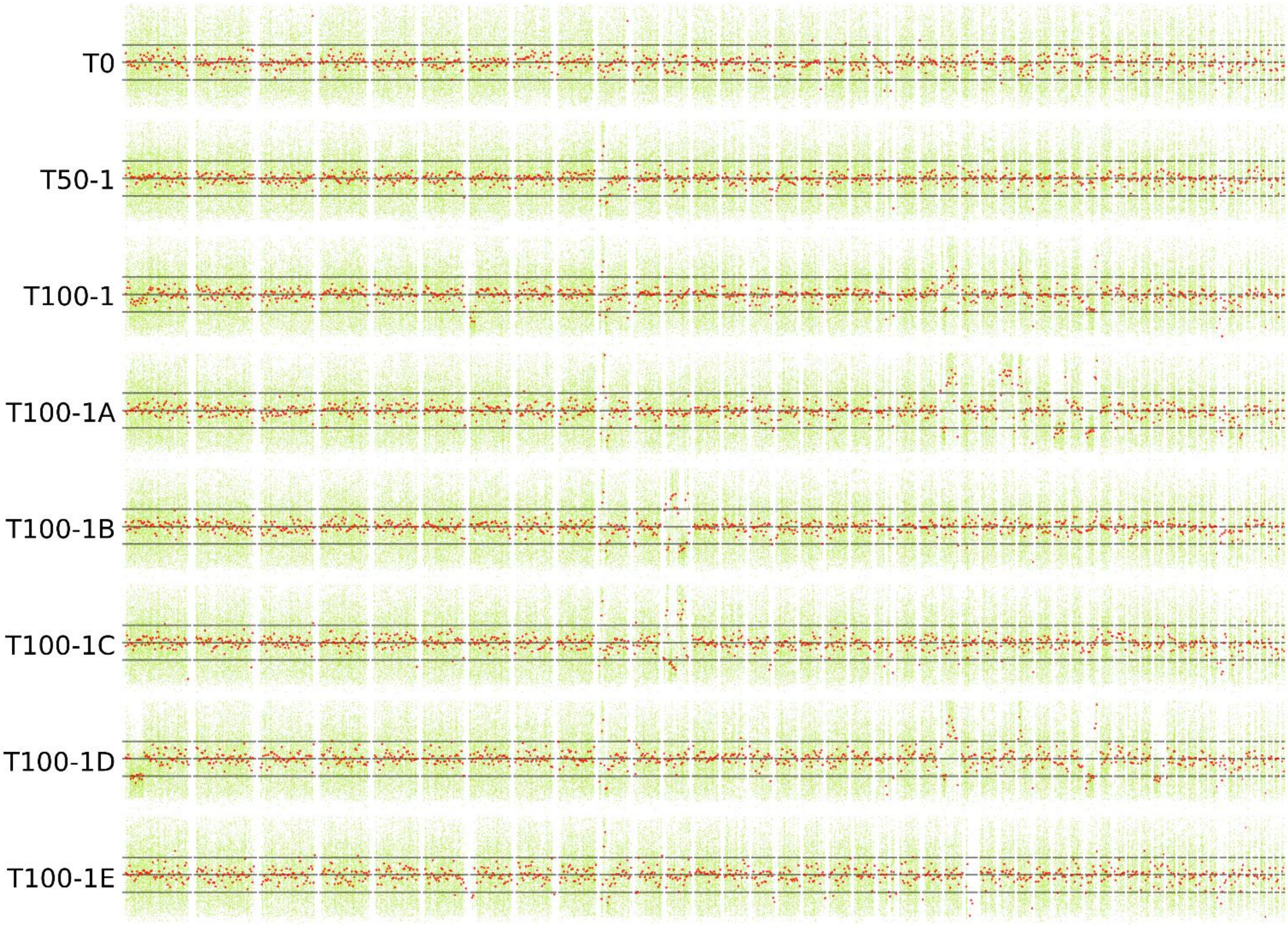

Supplemental Figure S1: Allelic ratio of heterozygous biallelic loci in the progenitor and the evolved strains of *Hortaea werneckii*. Biallelic single nucleotide polymorphism loci were determined by comparing whole-genome sequencing reads to a haploid *H. werneckii* reference genome using the Genome Analysis Toolkit (GATK). The proportion of non-reference alleles was calculated and normalised by the median of the alternative allele proportion of the strain by subtracting the median from the proportion of the depth per-locus and dividing the result by the median. For the fifty largest contigs of the reference haploid genome, the arithmetic mean of proportion in 50 bp bins is shown in green, overlayed by the median proportion in 30 kbp bins (red). Horizontal grey lines mark the values 0 (approximately expected non-reference allelic ratio for diploid regions in a majority diploid genome), -0.33, and 0.33 (approximately expected non-reference allelic ratio for triploid regions).

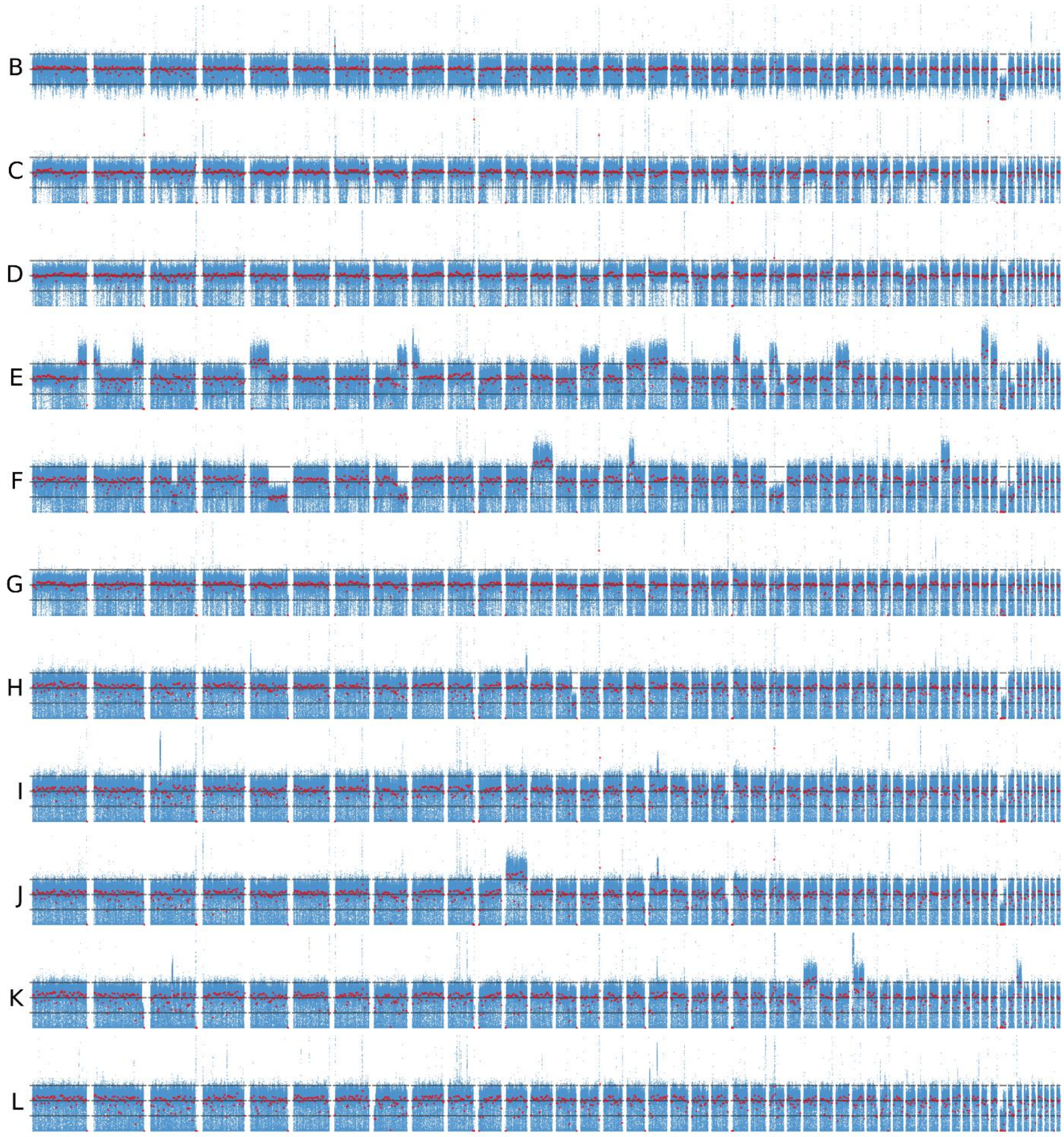

Supplemental Figure S2: Sequencing coverage of wild isolates of *Hortaea werneckii*. Whole-genome sequencing reads were mapped to a haploid *H. werneckii* reference genome. The per-nucleotide sequencing depth was normalised with the median sequencing depth of the strain by subtracting the median from the per-nucleotide depth and dividing the result by the median. For the fifty largest contigs of the haploid reference genome, arithmetic mean coverage in 50 bp bins is shown in blue, overlayed by the median coverage in 30 kbp bins (red). Horizontal lines mark the values 0 (approximately expected coverage for diploid regions in a majority diploid genome), -0.5 (approximately expected coverage for haploid regions), and 0.5 (approximately expected coverage for triploid regions). In case of haploid strains C and D the value 0 represents haploid regions of the genome.

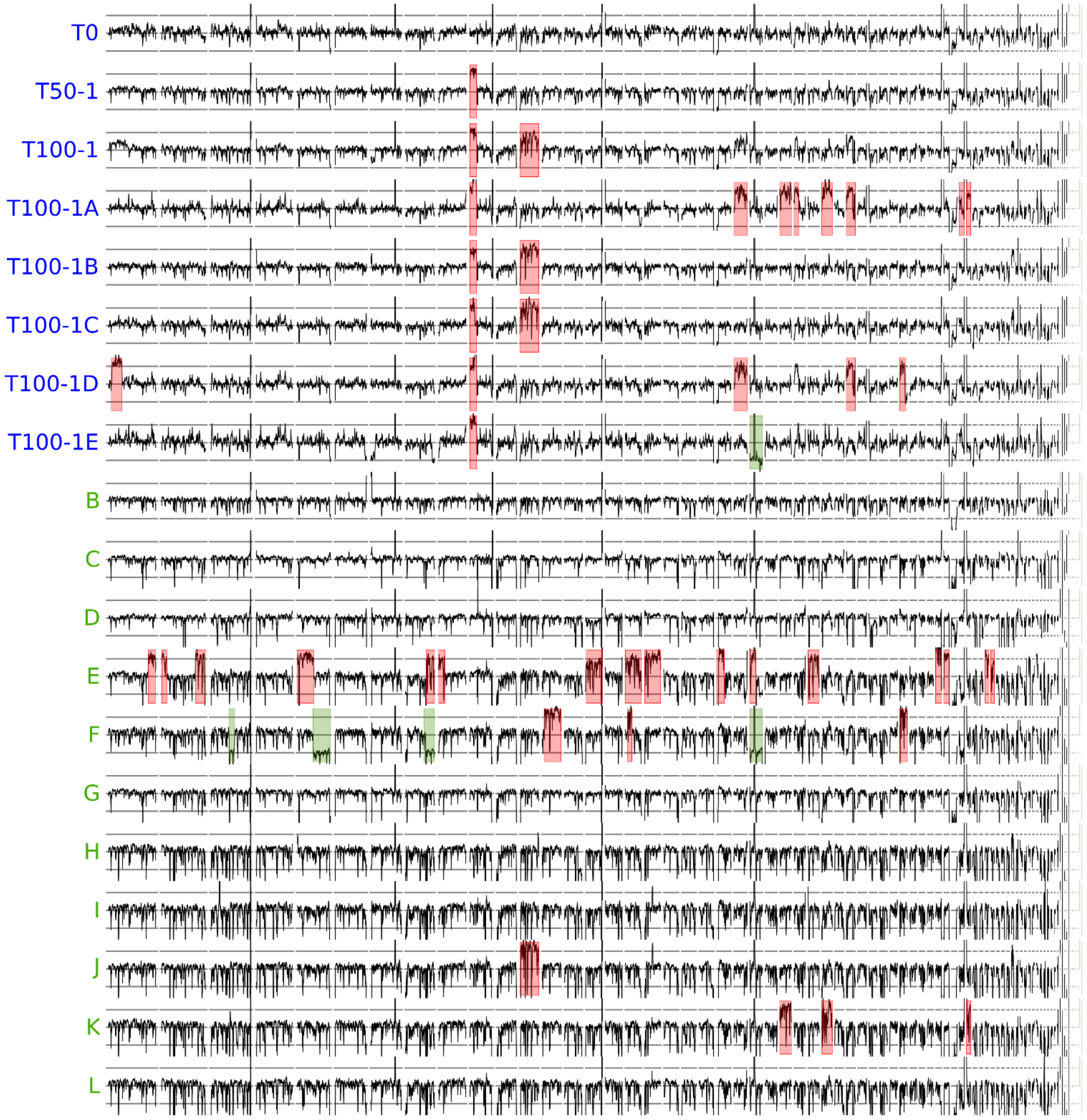

Supplemental Figure S3: Sequencing coverage and aneuploid regions of *Hortaea werneckii* genomes. Progenitor strain (T0), evolved strains (T50-1 to T100-1E), and wild strains (B to L) of H. werneckii are shown in rows. Rolling median of sequencing coverage calculated in 20 kbp windows along the contigs of the haploid reference genome of *H. werneckii* (black line). Computationally identified triploid regions are indicated with red rectangles, and haploid regions are indicated with green rectangles.
